# Genetic polymorphism of merozoite surface protein-1 in *Plasmodium falciparum* isolates from patients with mild to severe malaria in Libreville, Gabon

**DOI:** 10.1051/parasite/2015012

**Published:** 2015-03-18

**Authors:** Marielle Karine Bouyou-Akotet, Noé Patrick M’Bondoukwé, Denise Patricia Mawili-Mboumba

**Affiliations:** 1 Département de Parasitologie-Mycologie, Faculté de Médecine, Université des Sciences de la Santé Libreville Gabon; 2 Unité de Recherche Clinique et Opérationnelle sur le Paludisme, Hôpital Régional de Melen BP 4009 Libreville Gabon

**Keywords:** *P. falciparum*, Symptomatic malaria, Age, *msp1*, Gabon

## Abstract

We assessed *Plasmodium* (*P.*) *falciparum* allelic diversity based on clinical severity and age. The study was conducted from 2011 to 2012 in Libreville, Gabon where malaria prevalence was 24.5%. The polymorphism of the *merozoite surface protein-1* (*msp1*) locus was analyzed in isolates from patients with complicated and uncomplicated malaria. Blood was collected on filter paper. After DNA extraction, genotyping of the *msp1* gene was performed using nested PCR. The K1, Ro33, and Mad20 allelic families were detected in 71 (63%), 64 (57%), and 38 (34%) of the 112 analyzed samples, respectively. Overall, 17 K1 and 11 Mad20 alleles were detected. There was no association between *msp1* allelic families and age. Mad20 allelic diversity increased with the severity of malaria. The number of K1 and Mad20 alleles decreased with age. The multiplicity of infection (MOI) was 1–6 genotypes and the complexity of infection (COI) 1.8 ± 1. The COI differed based on age: it was 1.9 (±1.1) in the isolates from adults, 1.8 (±1.1) in those from 0–5 year-old children, whereas it tended to be lower (1.6 ± 0.8) in those from 6–15 year-old children. Extensive genetic diversity is found in *P. falciparum* strains circulating in Libreville. The number of specific *msp1* alleles increased with clinical severity, suggesting an association between the diversity and the severity of malaria.

## Abstract (fr)

Nous avons analysé la diversité allélique de *Plasmodium falciparum* en fonction des formes cliniques du paludisme et de l’âge. L’étude a été conduite de 2011 à 2012 à Libreville (Gabon) où la prévalence du paludisme était de 24.5 %. Le polymorphisme du gène *merozoite surface protein 1* (*msp-1*) a été étudié dans des isolats de patients présentant un paludisme simple ou grave. Le sang a été recueilli sur du papier buvard. Aprés extraction de l’ADN, le gène *msp1* a été analysé par PCR nichée. Les familles alléliques K1, Ro33 et Mad20 ont été détectées dans respectivement 71 (63 %), 64 (57 %) et 38 (34 %) des 112 échantillons analysés. Au total, 17 allèles K1 et 11 allèles Mad20 ont été détectés. Aucune association entre une famille allélique donnée de *msp-1* et l’âge n’a été mise en évidence. La diversité allélique de la famille Mad20 augmente avec la sévérité de la maladie. Le nombre d’allèles différents de K1 et Mad20 diminue avec l’âge. La multiplicité des infections (MOI) était de 1 à 6 et la complexité des infections (COI) de 1.8 (± 1). La COI variait en fonction de l’âge, elle était de 1.9 (± 1.1) dans les isolats des adultes, de 1.8 (± 1.1) dans ceux des enfants de 0–5 ans, tandis qu’elle avait tendance à être plus faible (1.6 ± 0.8) dans ceux des enfants de 6–15 ans. La diversité génétique des souches de *P. falciparum* recueillies à Libreville est importante. Le nombre d’allèles spécifiques du gène *msp-1* augmente avec la sévérité clinique, suggérant une association entre la diversité et la sévérité du paludisme.

## Introduction

Malaria is the most significant of the parasitic diseases, affecting 198 million people worldwide [39]. The outcome of *Plasmodium* (*P.*) *falciparum* infection is variable, ranging from asymptomatic to complicated malaria [27]. Parasite virulence contributes directly to the clinical outcome and parasite diversity influences the speed at which strain-specific immunity develops in the host population. In areas of perennial and intense malaria transmission, antimalarial immunity develops with age, exposure, and frequency of different *P. falciparum* infections. The consequence is a decrease of severe malaria cases with increasing age [17, 35]. *P. falciparum* infections often consist of genetically distinct populations, i.e. clones of the same parasite species [26, 30]. Clone competition may affect host morbidity and transmission, and influences parasite virulence and drug resistance [24, 29]. However, it is not well understood whether severe malaria is due to binding to multiple receptors from a single population of parasites or to several infecting clones binding to different specific receptors [5, 16]. Recently, domain cassettes 8 and 13 encoded by *var* genes that are associated with severe malaria were identified [18].

Between 2003 and 2011, an epidemiological transition of malaria prevalence was observed in Gabon. A rebound of clinical malaria prevalence in the main cities and a shift toward a higher frequency of malaria infection and morbidity in older children and adults who are thought to have acquired premunition, has also been observed [9, 22]. One explanation could be exposure to particular genotypes as well as multiplicity of infections (MOI) that have been described as different according to transmission intensity and to the outcome of infection [3, 29]. Comparative analysis of parasite genetic characteristics in isolates collected from mild and severe malaria showed a particular *msp1* block 2 allele B-K1 and a particular *var* gene (var-D) in isolates from patients with severe *P. falciparum* malaria [5, 19]. Merozoite surface proteins (MSPs) are the most commonly used markers of *P. falciparum* genetic diversity because of their high polymorphism. Some genotypes are thought to be more or less virulent because of their predominance in severe malaria or asymptomatic cases [5, 34]. Amodu et al. found that the presence of variant alleles from the K1 and Mad20 allelic families was associated with a lower risk of developing malaria in asymptomatic patients [3]. Thus, analysis of the *msp1* gene and its variants in infected individuals with different clinical forms in Gabon could contribute to knowledge of parasite virulence patterns associated with disease susceptibility based on age. Moreover, it will provide updated data, since those on the frequency of multiplicity of infections (MOI) and *P. falciparum* genetic diversity using *msp1* were obtained with samples collected more than 10 years ago [16, 23, 38]. The aim of this study was to determine whether the clinical manifestations of malaria are associated with a particular allelic family *msp1* genotype in Gabon, Central Africa.

## Patients and methods

### Study site and population

Data were collected during prospective cross-sectional surveys conducted in 2011–2012 in Gabon, at the Centre Hospitalo-Universitaire de Libreville (CHL) and the Regional Hospital of Melen (RHM), two sentinel sites for malaria surveys selected by the Malaria National Control Program (MNCP). The CHL is located in Libreville, the capital city, and the RHM of Melen in a suburban area situated 11 km north of Libreville. These study sites were previously described elsewhere [9, 22]. In sentinel sites, screening of febrile patients for malaria and monitoring of molecular markers for *P. falciparum* drug resistance are routinely performed. Therefore, data collection was part of routine activities at the CHL and RHM. The prevalence of clinical malaria among febrile patients consulting at these two health centers was 24.1% and 31.3% in 2011–2012, respectively [22]. Febrile *P. falciparum*-infected outpatients and inpatients were included after their acceptance to participate in the study. Oral informed consent was obtained to complete the demographic and medical history sections of a case report form (CRF). Tympanic temperature and clinical symptoms leading to the consultation and/or hospitalization were recorded on the CRF.

### Malaria diagnosis

Thick films were performed as previously described [22]. They were dried and stained with 20% Giemsa for 20 min. Microscopic reading of 100 oil immersion fields was performed and parasitemia was expressed as the number of parasites per microliter of blood. Smears were considered negative when the examination of 100 oil immersion fields did not reveal any parasite. Thin blood smears were used for species identification.

### DNA extraction and *msp1* amplification

DNA was extracted from peripheral blood collected in EDTA tubes using the QIAamp^®^ kit (QIAGEN^®^) according to the manufacturer’s instructions. The polymorphic region of block 2 of *msp1* was amplified by nested PCR using the protocol described by Ntoumi et al. [28]. Briefly, for the first reaction, primer pairs of the conserved sequence spanning the *msp1* block 2 region were used: the product generated in this reaction served as a template in the second reaction, performed with a primer pair allowing allelic variant identification of the K1, Mad20, and Ro33 *msp1* Block 2 families. Each genotype was identified based on the size of the PCR products using 2% agarose gel electrophoresis.

### Definitions

According to the Newton and Krishna classification, malaria patients were classified into three groups: (1) severe malaria (SM) patients who strictly met the WHO 2000 criteria for severe malaria; (2) moderate malaria (MM) patients who did not have any clinical or biological features of SM according to WHO classification, but required parenteral treatment and hospitalization because of symptoms such as asthenia, vomiting, convulsions; and (3) uncomplicated malaria (UM) patients who were febrile outpatients without features of severe or moderate disease and who were able to be treated with oral medication [27].

Detection of a single PCR fragment was classified as monoclonal infection and that of more than one fragment as multiple infection. The frequency of single infection was the number of patients with one parasite genotype over the total infected population. The complexity of infection (COI) was defined as the average number of different genotypes per infected patient.

### Ethical considerations

The study objectives were clearly explained to the ward staff. Patients were informed about the protocol and their oral consent was required prior to data collection. Although this was a non-invasive study, oral consent sought to obtain medical history and to use demographic, clinical, and biological data. Data were kept confidential.

The study was approved by the Ministry of Health. As a reference laboratory for the MNCP, the Department of Parasitology and Mycology has the health authority’s approval to monitor the evolution of malaria morbidity throughout the country through sentinel sites. Malaria diagnosis was free of charge and infected patients were treated according to national policy. For patients aged less than 18 years, individual oral assent and oral consent of legal guardians were required prior to inclusion.

### Statistical analysis

Statview software version 5.0 (SAS Institute Inc) was used for data analysis. Differences between groups were assessed using chi-squared or Fisher’s exact tests for proportions, Student’s *t*-test and analysis of variance (ANOVA) or the Kruskal-Wallis test as appropriate for continuous variables. A *p*-value less than 0.05 was considered significant.

## Results

Overall, samples from 112 infected patients were analyzed. Patients were classified by age as follows: 0–5 years (*n* = 55), 6–15 years (*n* = 25), and over 15 years (*n* = 32). One third of patients had severe malaria (42%; *n* = 47/112). Based on clinical status, the distribution of male patients was comparable (Table 1). Median parasitemia was higher among patients with severe malaria (*p* < 0.01) (Table 1). Adults were more frequently in the group of patients with moderate malaria (46%; *n* = 27/58) and all patients with severe malaria were aged less than 6 years.

**Table 1.** Patients characteristics.

	Mild malaria	Moderate malaria	Severe malaria	*P*
*N*	38	27	47	
Mean age, months (±SD*)	149.9 (±113.3)	270.5 (±192.6)	39.5 (±29.6)	<0.01
Male, *n* (%)	12 (32.1)	14 (51.9)	22 (46.8)	>0.05
Median parasitemia (*p*/µL)	24,746	60,946	85,282	<0.01

* Standard deviation.

### Frequency of the different *msp1* allelic families depending on clinical status and age

K1, Ro33, and Mad20 allelic families were detected in 63.4% (*n* = 71/112), 57.1% (*n* = 64/112), and 33.9% (38/112) of the isolates, respectively. K1-type alleles were frequently detected in isolates whatever the clinical status (≥60%) (*p* < 0.01) (Table 2). Ro33 variants were predominant in isolates from patients with severe malaria (*n* = 30; 64%), however no statistical difference was found (*p* = 0.27) (Table 2). Likewise, age was not associated with distribution of each allelic family (*p* > 0.1). The distribution of the Mad20 allelic family was comparable based on clinical status (*p* = 0.72) and age (*p* = 0.63) (Table 2).

**Table 2.** *Msp-1* allelic family distribution according to clinical status and age.

	Malaria clinical forms				Age groups			
	Mild malaria (*N* = 38)	Moderate malaria (*N* = 27)	Severe malaria (*N* = 47)	*p*	0–5 years (*N* = 55)	5–15 years (*N* = 25)	Adults (*N* = 32)	*p*
K1, *n* (%)	25 (66)	17 (63)	29 (62)	0.92	36 (65)	17 (68)	18 (56)	0.60
Ro33, *n* (%)	22 (58)	12 (44)	30 (64)	0.27	29 (53)	13 (52)	22 (69)	0.29
Mad20, *n* (%)	11 (29)	10 (37)	17 (36)	0.72	17 (31)	8 (32)	13 (41)	0.63

### K1 and Mad20 allelic diversity, clinical status and age

**Figure 1.**
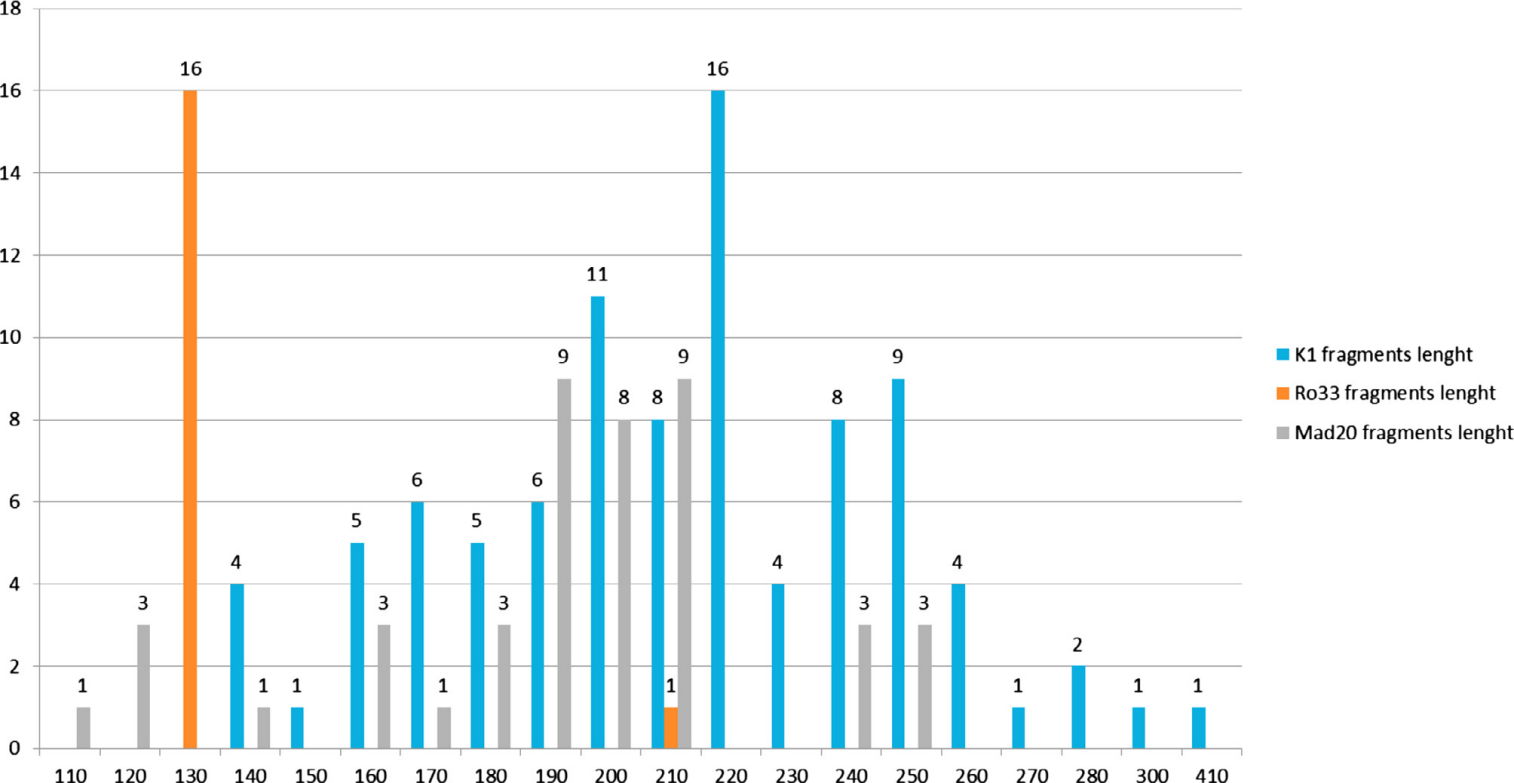
K1, Ro33, and Mad20 allele distribution according to their length (base pairs).

In total, 17 different K1-type alleles were detected, with the K1-220 allele being found most frequently (24%; *n* = 16/71) (Fig. 1). Among the Mad20-type alleles (*n* = 11), the main alleles were Mad20-190 (24%; *n* = 9/38) and Mad20-210 (24%; *n* = 9/38). Two Ro33 alleles were detected, one of 210 bp was found in one isolate, while 63 other isolates carried the Ro33-130 allele (Fig. 1).

**Figure 2.**
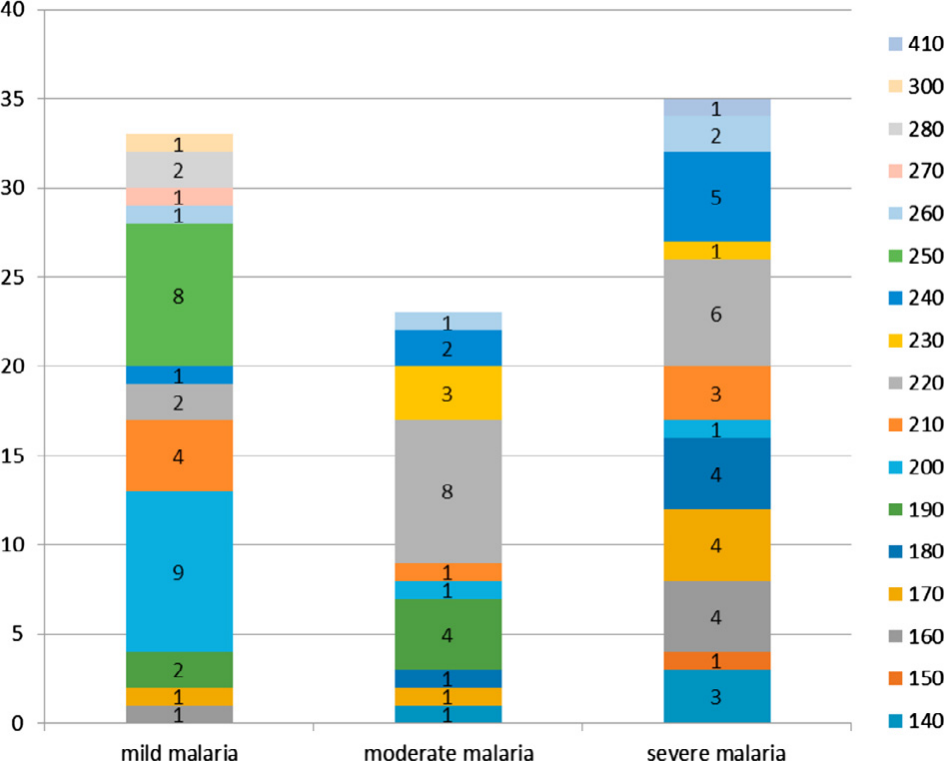
K1 allele distribution according to malaria clinical forms.

Among the different K1 alleles detected, more than 70% were found in isolates from patients with severe (*n* = 12/17) and mild malaria (*n* = 12/17) (Fig. 2). The K1-200 allele predominated in isolates from patients with mild malaria (82%; *n* = 9/11), the K1-220 allele in those from patients with moderate or severe malaria, and none of the severe malaria patients were infected by K1-190 (Fig. 2). Four alleles (K1-250, K1-270, K1-280, and K1-300) were specifically found in isolates from patients with mild malaria, while K1-240 was mainly detected in severe malaria patients (*n* = 5/8) and mild malaria patients (*n* = 2/8).

**Figure 3.**
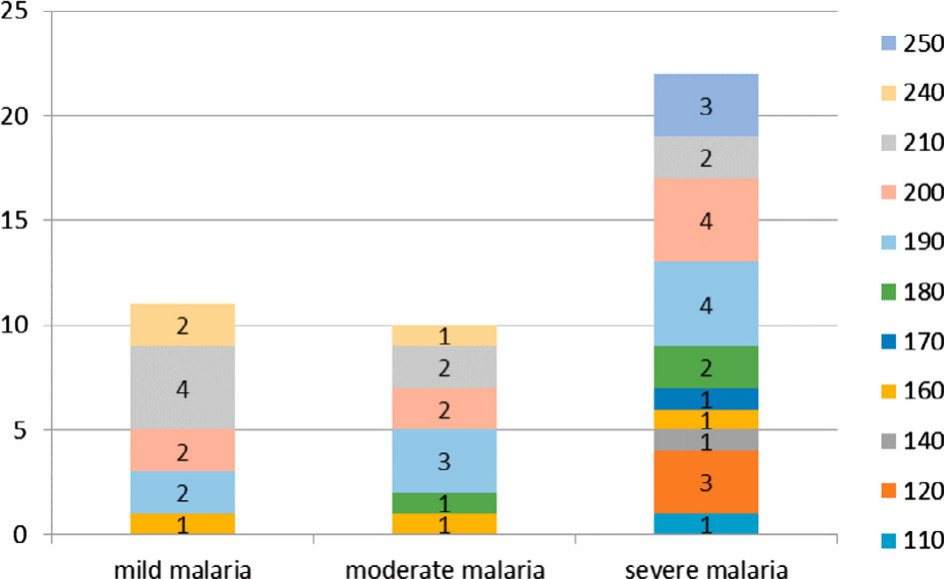
Mad20 allele distribution according to malaria clinical forms.

Mad20 allelic diversity increased with the severity of malaria: 45.4% (*n* = 5/11) and 91% (*n* = 10/11) of the different alleles were found in isolates from patients with mild malaria and severe malaria, respectively. All Mad20 variants (45.4%; *n* = 5/11) found in isolates from patients with mild malaria were also detected in those from patients with moderate malaria. The frequencies of Mad20-200 and Mad20-190 alleles increased with disease severity while lower frequency of the Mad20-210 genotype was found in isolates from patients with severe and moderate malaria compared to those with mild malaria (Fig. 3).

**Figure 4.**
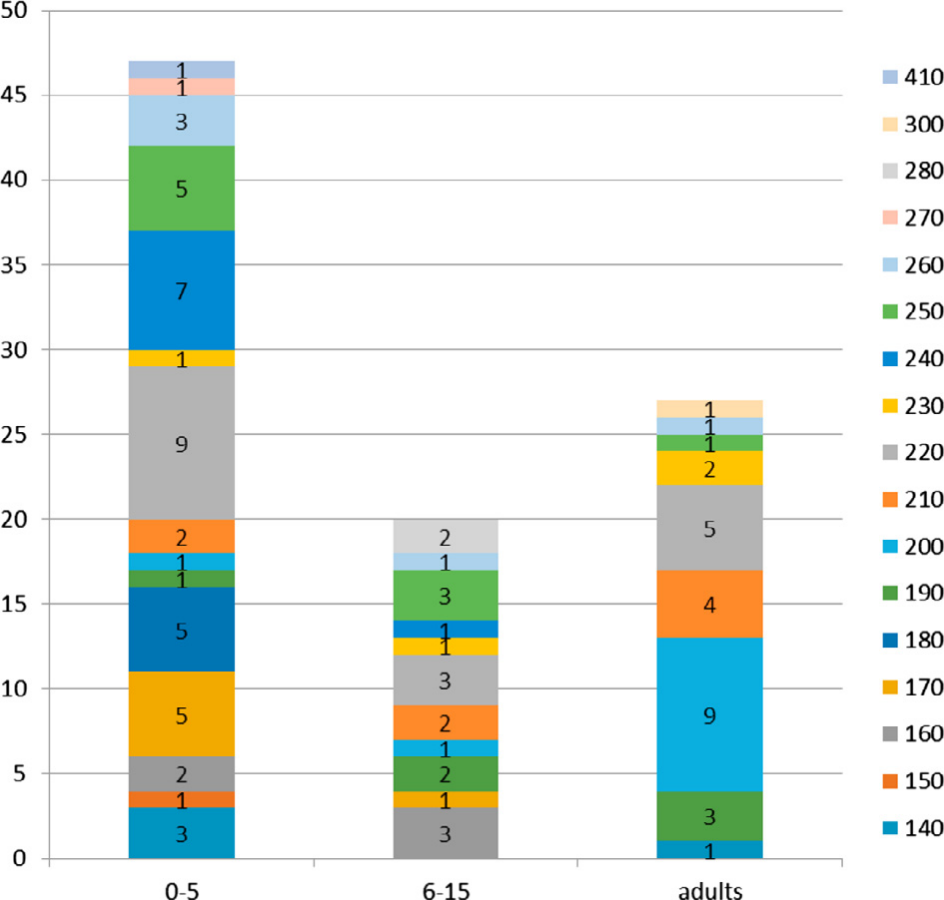
K1 allele distribution according to age groups.

**Figure 5.**
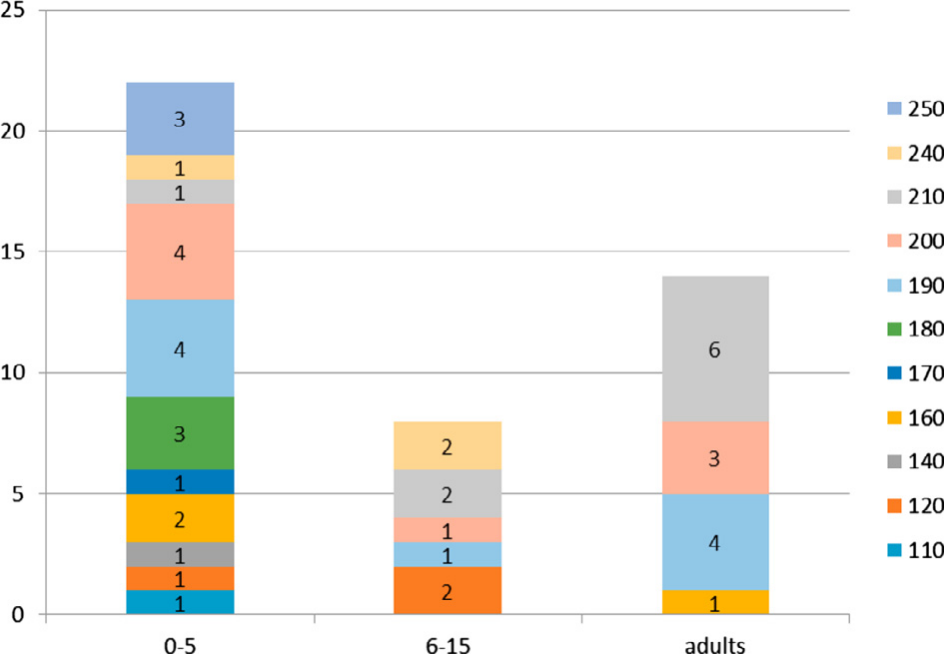
Mad20 allele distribution according to age groups.

The number of K1 and Mad20 alleles decreased as a function of age; 88.2% (*n* = 15/17) and 53% (*n* = 9/17) of K1 alleles were detected in isolates from patients aged 0–5 years and adults, respectively (Figs. 4 and 5). K1-170 (83.3%; *n* = 5/6) and K1-240 (87.5%; *n* = 7/8) alleles were frequently found in the youngest patient isolates; five of them had severe malaria. These alleles were not detected among adults. K1-190 was mostly found in patients older than 5 years (*n* = 5/6), and K1-200 in isolates from adults (82%; *n* = 9/11) (Fig. 4). All Mad20 alleles (*n* = 11) were detected in isolates from the 0–5 year children group and only in four from adults (Fig. 5). Mad20-110, Mad20-140, Mad20-170, Mad20-180, and Mad20-250 alleles were specifically identified in isolates from patients aged less than 6 years (Fig. 5). The Mad20-190, Mad20-250, and Mad20-210 alleles that were predominantly identified in severe malaria patients were also found in infected adults.

Half of the infections were monoclonal (50%; *n* = 56/112). The monoclonal infection rates were comparable between patients with mild (53%; *n* = 20/38), moderate (52%; *n* = 14/27), and severe malaria (47%; *n* = 22/47). Based on age, monoclonal infection proportions did not vary between patients aged 0–5 years (53%; *n* = 29/55) and 6–15 years (52%; *n* = 13/25). On the contrary, adults more frequently carried polyclonal infections (56%; *n* = 18/32). However, the distribution of simple and multiple infections was not statistically different according to the clinical presentation and age (*p* > 0.05).

One to six clones were detected per isolate. The complexity of infection (COI) was 1.82 ± 1.04; it was comparable between the three clinical groups (1.7, 1.9 and 1.8 in moderate, severe, and mild malaria patients, respectively) (*p* > 0.05). The COI of adult isolates (1.94 ± 1.08) was higher compared to that for 6–15 year-old children (1.64 ± 0.76), although statistical analysis did not reach significance (*p* = 0.07). However, the COI of the children aged 0–5 years (1.84 ± 1.13) was higher than the COI of older children (*p* = 0.02).

## Discussion

Analysis of the *P. falciparum* genetic profile according to the clinical severity of malaria may provide useful information on specific parasite characteristics to design intervention strategies targeting virulence factors [13]. It has been shown that most alleles fluctuate significantly over the years and can differ across endemic areas [14, 40]. The present study provides for the first time, the genetic diversity of *P. falciparum* isolates from Libreville, where 40% of Gabon’s population lives. Previous data were obtained more than 10 years ago in two other cities, Lambaréné and Franceville [6, 12, 16].

Extensive genetic polymorphism within the *msp1* allelic families (30 alleles identified) is observed in isolates from Libreville. This is consistent with the diversity found in Bakoumba near Franceville (25 alleles) in 1999, in Dielmo, Senegal (33 alleles) in 1995, and in Mauritania (27 alleles) in 2010 [1, 6, 15]. In contrast, only nine alleles were found in Franceville in 1999 and a higher diversity (41 alleles) was observed in Burkina Faso in 2009, even though these areas display comparable malaria endemicity patterns [12, 36]. The study revealed the predominance of the K1 allelic family as previously observed in Gabon, Benin, and Nigeria [12, 30, 32]. The Ro33 allelic family was more frequently detected compared to Mad20, in contrast to data from Uganda and Mauritania [1, 14].

Overall, 17 and 11 different alleles were found in the K1 and Mad20 families, respectively, whereas the Ro33 allelic family was poorly polymorphic. The number of alleles is comparable to that found in isolates collected in Bakoumba (14 K1-type alleles, 8 Mad20-type alleles, and 3 Ro33-type alleles), but higher than that found in isolates from Franceville, located 110 km from Bakoumba, where six different K1-type, two Ro33-type, and one Mad20-type alleles were detected [6, 12]. This high diversity could partly contribute to the change of malaria prevalence observed in Libreville. In fact, after the decrease of malaria morbidity which followed the implementation of intermittent preventive treatment during pregnancy, insecticide-treated bed nets and artemisinin-based combination therapies in Gabon, a rebound of malaria cases was observed, mainly among older children and adults who are thought to have acquired premunition. The most frequently encountered clinical forms are mild and moderate malaria in these populations [8, 9, 22].

The existence of new circulating parasite strains specific to different clinical outcomes should thus be investigated. Although previous data that would allow the comparison of the level of genetic diversity in Libreville are lacking, frequent genetic recombinations between the *msp1* gene could lead to the appearance of novel alleles in high transmission areas such as Libreville [37]. Thus, older children and adults may be infected by new parasite strains against which they do not have protective immunity.

A high frequency of Ro33 alleles was reported to be associated with the less severe disease presentation: asymptomatic and mild malaria [2, 16]. In Nigeria, the absence of both the K1 and Mad20 allelic families was a risk factor for developing clinical manifestations among asymptomatic subjects. In Tanzania, the presence of the Mad20 allelic family was associated with fever [3, 21]. In our study, the distribution of the K1, Ro33, and Mad20 allelic families was not related to any clinical form. Moreover, Ro33 alleles predominated among patients with severe malaria, although K1 and Mad20 allelic diversity was the highest in isolates collected from patients with severe malaria. Some alleles were predominant in moderate and severe malaria groups. Indeed, frequencies of K1-220, K1-180, K1-170, Mad20-200, and Mad20-190 increased with disease severity; they were also detected less in isolates from mild malaria patients in which K1-240, K1-200, and Mad20-210 predominated. This genetic pattern suggests an association between malaria severity and some parasites with specific “virulence”. Ariey et al. also showed the overexpression of specific *msp1* and *var* gene alleles among patients with severe malaria [5]. Moreover, the K1-250 allele was specific to the isolates from patients with mild malaria and the Mad20-250 allele was specific to the severe malaria group. In a study performed in Uganda, no association was found between a particular *msp1* genotype and disease severity [14]. The use of other markers such as microsatellites highlighted greater genetic diversity in isolates from patients with severe malaria, some alleles (2–3) predominated at particular loci and were specifically related to the clinical outcome [7]. Some specific parasite strains could have been missed because of results underpowered by the small number of analyzed isolates in the present study. It is also known that simultaneous analysis of multiple loci would provide more indications on the parasite genetic diversity. Nevertheless, it was shown that the study of allelic diversity using one *msp1* locus is able to provide important and interesting information, particularly according to age and clinical status, as presented here [19, 29, 37]. Furthermore, parasite specific antibodies against the block 2 of *msp1* are associated with malaria acquired immunity [11]. Antibody acquisition against different parasite strains is necessary to protect against progression from an asymptomatic infection to clinical disease [12, 29]. This acquired immunity is associated with control of parasite density. It is generally accepted that immunity to malaria develops with age after exposure to different *P. falciparum* infections. The ability to clear the parasite is associated with the development of antimalarial immunity directed against several genetically different *P. falciparum* strains [10]. If an allelic family is predominantly found in one infected population, one can assume that subjects will develop immunity against these alleles and the presence of less frequent genotypes will more easily induce symptoms. Allelic diversity decreased with age of the patients. Different K1 and Mad20 alleles were mainly detected among the youngest patients, while almost half of the alleles or less could be detected among adults. The K1-180, Mad20-180, and Mad20-250 alleles were solely found among the youngest children (0–5 year group). None of these detected alleles was specifically found in the group of patients with moderate malaria and those aged 6–15 years.

Some alleles, Mad20-210, Mad20-200, Mad20-190, Mad20-160, and K1-200, were identified in adults with uncomplicated malaria while they predominated in isolates from children with severe malaria symptoms. Thus, the low level of premunition in young children may impede their capacity to control the infection by these strains. The same suggestion can be proposed for the K1-240 alleles which are not found among adults and caused severe malaria in the majority of children under 6 years of age.

The mean number of *msp1* genotypes per individual was 1.8, as found previously in Franceville, Bakoumba, and Congo Brazzaville, but it was lower than the COI calculated in other endemic areas such as in Benin and Burkina Faso [6, 12, 23, 30, 36]. The complexity of infections was similar in isolates from patients based on the clinical status in contrast to data reported by others [7, 14]. Adults generally carried a higher number of clones (COI 1.94) compared to the youngest children (COI varied between 1.64 and 1.84). In highly endemic areas, individuals developed premonition with age and, through perennial exposure to mosquito bites, they maintain their immunity. The number of clones co-infecting a single host can be used as an indicator of the level of malaria transmission or the level of host acquired immunity that is related to the endemicity [4, 33]. Indeed, the mean number of parasite clones per host was shown to increase with the level of malaria transmission or endemicity; the high polymorphism of *P. falciparum* circulating isolates is in favor of increased malaria transmission in the city as superinfections with multiple parasite genotypes were frequent, together with within-host competition in circulating parasites from high endemic settings [4, 24].

The proportions of patients carrying multiple infections are comparable according to age, suggesting that aside from a lack of or reduced premunition, other factors may contribute to the increased susceptibility to malaria in individuals of all ages living in Gabon. It was recently reported in Benin and Nigeria that MOI was not influenced by age [30, 31]. Host factors, such as gender, antimalarial drug use, and co-infection with intestinal and blood parasites, influence the clinical outcome [7, 20]. Variations in rainfall abundance and seasonality also influence parasite genetic factors [24]. During *P. falciparum* infection with several genotypes, there is selection for effective transmission of sexual gametocyte stages to mosquitoes, probably as a result of the presence of specific alleles which mediate the survival of the parasite inside the mosquito, thus increasing the probability of infected mosquito vector abundance and the successful parasite transmission to humans through a high number of infectious bites [20, 24, 25]. Studies analyzing the relationship between entomological inoculation rates, the multiplicity of infection, and genetic diversity of circulating parasites in areas with different levels of endemicity, should be performed.

A direct relationship between disease severity and the frequency of specific alleles detected was not found, probably due to the small number of patients within each group. The use of nested PCR instead of microsatellites or sequencing would have underestimated genetic diversity. Indeed, it is known that daily changes in parasite genotypic patterns occur due to parasite sequestration. Alleles of identical size with point mutations as well as those from additional genotypes that are present beside the predominant ones at low density and low frequency at the time of blood sampling, would not have been detected by nested PCR or well identified after agarose gel electrophoresis. However, useful and accurate data are here provided.

## Conclusion

Based on *msp1* gene genotyping, high parasite genetic diversity is observed in *P. falciparum* strains circulating in Libreville. An increasing number of K1 and Mad20 alleles with increasing age and disease severity was observed. Moreover, specific alleles were found among the youngest patients and the adults. Indeed, some K1 and Mad20 alleles tend to be related to disease severity among children and to uncomplicated malaria among adults.
